# First detection and genotyping of *Enterocytozoon bieneusi* in reindeers (*Rangifer tarandus*): a zoonotic potential of ITS genotypes

**DOI:** 10.1186/s13071-015-1155-0

**Published:** 2015-10-12

**Authors:** Weishi Liu, Chunyu Nie, Longxian Zhang, Rongjun Wang, Aiqin Liu, Wei Zhao, Heping Li

**Affiliations:** College of Wildlife Resources, Northeast Forestry University, Harbin, Heilongjiang 150040 China; College of Animal Science and Veterinary Medicine, Henan Agricultural University, Zhengzhou, Henan 450002 China; Department of Parasitology, Harbin Medical University, Harbin, Heilongjiang 150081 China

**Keywords:** *Enterocytozoon bieneusi*, ITS gene, Genotying, Reindeers, Zoonotic

## Abstract

**Background:**

*Enterocytozoon bieneusi* is the most common pathogen of 14 microsporidian species infecting humans worldwide. In China, *E. bieneusi* has been reported in some common livestock and environmental specimens. However, no information is available on occurrence of *E. bieneusi* in reindeers. The objective of the present study was to detect and genotype *E. bieneusi* in reindeers in China, and assess the zoonotic potential.

**Findings:**

125 fecal specimens were collected from wild reindeers in the northeast forest region of Great Hinggan Mountains of China. By PCR and sequencing of the internal transcribed spacer (ITS) region of the ribosomal RNA (rRNA) gene of *E. bieneusi*, an average infection rate of 16.8 % (21/125) was observed in reindeers. *E. bieneusi* was detected in two age groups: 7.7 % (3/39) in the youths (aged 1 to 2 years) and 22.2 % (18/81) in the adults (aged 3 to 8 years). Five genotypes were identified: one known genotype Peru6 (*n* = 6) and four novel genotypes named as CHN-RD1 (*n* = 12), and CHN-RD 2 to CHN-RD4 (one each). In phylogenetic analysis, all the novel genotypes together with known genotype Peru 6 were clustered into group 1.

**Conclusions:**

This is the first report of *E. bieneusi* infection in reindeers, expanding the host range of *E. bieneusi*. The fact of genotype Peru 6 previously reported in humans and the result of all the novel genotypes falling into zoonotic group 1 suggest the possibility of *E. bieneusi* transmitted from reindeers to humans.

## Findings

### Background

Microsporidia are obligate intracellular eukaryotic pathogens composed of about 1300 species in 160 genera, and they have the ability to infect almost all animal phyla [1]. To date, at least 14 microsporidian species in eight genera have been described as human pathogens. *Enterocytozoon bieneusi* is the most frequently diagnosed species of microsporidia in humans [2]. Microsporidiosis caused by *E. bieneusi* is mainly characterized by chronic diarrhea and wasting in HIV-infected patients, but it appears to be asymptomatic or self-limited diarrhea in immunocompetent persons [3]. *E. bieneusi* is also common inhabitants of the gastrointestinal tract of a wide range of animal hosts, including mammals, birds and reptiles [4, 5].

Application of PCR-based molecular tools for genotyping *E. bieneusi* has contributed to a better understanding of the characteristics of this pathogen about its host specificity and transmission patterns. Due to the fact of a hypervariable sequence (243 bp) in the internal transcribed spacer (ITS) region of the ribosomal RNA(rRNA) gene within *E. bieneusi*, sequencing of the ITS gene is the standard method for genotyping *E. bieneusi* isolates [6]. Molecular data has shown that *E. bieneusi* is a complex species with multiple genotypes [2]. To date, at least 220 genotypes of *E. bieneusi* have been described, 64 of which have been detected in humans [2, 7, 8]. 51.56 % (33/64) of human-pathogenic genotypes are also found in animals, supporting a presumption of a zoonotic possibility of *E. bieneusi* [4, 9]. Phylogenetic analysis indicates that 94 % of the identified ITS genotypes of *E. bieneus* are in a large group named as human-pathogenic group or group 1, and the remaining ones are clustered into several potentially host-adapted groups named as groups 2 to 8 [10, 11].

At present, molecular epidemiological studies of animal microsporidiosis are mainly from some common livestock, and limited reports involve wild animals [11–15]. In China, reindeers are one of vulnerable animal species and there are at most 700 reindeers alive. The aims of the present study were to determine the natural infection rate of *E. bieneusi* in reindeers and genotype *E. bieneusi* isolates by PCR and sequencing of ITS gene as well as assess the potential of zoonotic transmission by phylogenetic analysis.

## Methods

### Ethics statement

The present study was carried out in accordance with the Law of the People’s Republic of China on the Protection of Wildlife of 1989. The research protocol was reviewed and approved by the Research Ethics Committee of Henan Agriculture University. Before collecting fecal specimens, we contacted the managers of reindeers and obtained their permission to have their animals involved. No animals were injured during this procedure.

### Collection of fecal specimens

In October 2014, approximately 10 g of fecal specimens was collected from 125 wild reindeers in captivity from three farms in the northeast forest region of Great Hinggan Mountains (51°10′N, 121°74′E) (Table 1). All the fecal specimens were collected from the ground immediately after defecation by using a sterile disposable latex glove and then placed in a labeled sterile bag individually. To avoid duplicate sampling of animals, each individual was identified according to their ear tag, neck rope, body characteristics such as color and size. All the specimens were transported to our laboratory in a cooler with ice packs within 24 h and stored at 4 °C prior to being used in molecular biological characterizations. Meanwhile, the source, age, gender and health status of each animal was recorded at the time of sampling. The ages of the reindeers ranged from one to 11 years, with all of them showing no clinical signs of illness.

**Table 1:** Prevalence and distribution of *E. bieneusi* genotypes by geography in China

Location	No of examined	No of positive (%)	*E. bieneusi* genotypes	
			Known (n)	Novel (n)
Genhe	59	10 (16.9)	Peru6 (2)	CHN-RD1 (6), CHN-RD2 (1), CHN-RD3 (1)
Alongshan	41	8 (19.5)	Peru6 (3)	CHN-RD1 (5)
Jinhe	25	3 (12.0)	Peru6 (1)	CHN-RD1 (1), CHN-RD4 (1)
Total	125	21 (16.8)	Peru6 (6)	CHN-RD1 (12), CHN-RD2 (1), CHN-RD3 (1), CHN-RD4 (1)

### DNA extraction

Each fecal specimen was homogenized in distilled water, filtered through gauze and centrifuged at 1500 *g* for 10 min at room temperature, followed by a wash in distilled water three times. Genomic DNA was extracted from 200 mg of each processed specimen using a QIAamp DNA Mini Stool Kit (Qiagen, Hilden, Germany) according to the manufacturer’s recommended procedures. The eluted DNA was stored at −20 °C until its analysis with PCR.

### PCR amplification

All the DNA preparations were detected for the presence of *E. bieneusi* by nested PCR amplification of a 389 bp nucleotide fragment of the rRNA gene of *E. bieneusi* and the primers and the cycling parameters in nested PCR analysis were used as previously described by Buckholt et al. [16]. TaKaRa Taq DNA Polymerase (TaKaRa Bio Inc., Tokyo, Japan) was used for all the PCR amplifications. A negative control with no DNA added was included in all PCR tests. All the secondary PCR products were subjected to electrophoresis in a 1.5 % agarose gel and visualized by staining the gel with ethidium bromide.

### Nucleotide sequencing and analysis

All the secondary PCR products of expected size were directly sequenced with a set of primers used for the secondary PCR on an ABI PRISM 3730 XL DNA Analyzer by Sinogeno-max Biotechnology Co., Ltd. (Beijing, China), using the BigDye Terminator v3.1 Cycle Sequencing Kit (Applied Biosystems, Foster City, CA, USA). Sequence accuracy was confirmed by two-directional sequencing and by sequencing additional PCR products if necessary for some DNA preparations.

ITS gene sequences obtained in the present study were aligned with each other and reference sequences downloaded from GenBank database using the Basic Local Alignment Search Tool (BLAST) (http://blast.ncbi.nlm.nih.gov/Blast.cgi) and Clustal X 1.83 (http://www.clustal.org/) to determine *E. bieneusi* genotypes. If the sequences obtained were identical to those published in GenBank, they were considered to be known genotypes and given the first published name. If not, they were considered to be novel genotypes. All the genotypes were named based on 243 base pairs of the ITS gene region of *E. bieneusi* according to the established nomenclature system [6].

### Phylogenetic analysis

To present the diversity of all the genotypes obtained in the present study and to assess the genetic relationship of novel ones here to known ones, a comparison of the ITS region of all the sequences obtained here and reference sequences published in previous studies was made using the software Mega 5 (http://www.megasoftware.net/) by constructing a neighbor-joining tree, based on the evolutionary distances calculated by a Kimura 2-parameter model. The reliability of these trees was assessed using bootstrap analysis with 1000 replicates.

## Results and discussions

In the present study, 16.8 % of 125 reindeers were found to be infected with *E. bieneusi*, with the highest infection rate in Alongshan Farm (19.5 %; 8/41), followed by 16.9 % (10/59) in Genhe Farm and 12.0 % (3/25) in Jinhe Farm (Table 1). *E. bieneusi* was only found in two age groups, youths aged 1–2 years (7.7 %, 3/39) and adults aged 3–8 years (22.2 %, 18/81), while infection rate of 9.6 % (5/52) in males and 21.9 % (16/73) in females (Table 2). In general, the present average infection rate was higher than that of the white-tail deer in New York City, the USA (12.2 %, 6/49) [11], but was lower than those in the white-tail deer (32.5 %, 26/80) in Maryland, the USA [15], and in sika deer 32.6 % (28/86) and in red deer 20 % (1/5) in China [9].

**Table 2:** Prevalence and distribution of *E. bieneusi* genotypes by age and gender

Group	No of examined	No of positive (%)	*E. bieneusi* genotypes (n)
Age (year)			
<2	39	3 (7.7)	Peru 6 (1), CHN-RD1 (1), CHN-RD2 (1)
3-8	81	18 (22.2)	Peru 6 (5), CHN-RD1 (11), CHN-RD3 (1), CHN-RD4 (1)
>9	5	0	–
Gender			
Male	52	5 (9.6)	CHN-RD1 (4), CHN-RD2 (1)
Female	73	16 (21.9)	Peru 6 (6), CHN-RD1 (8), CHN-RD3 (1), CHN–RD4 (1)

The bars denote negative results at the locus

**Fig. 1:**
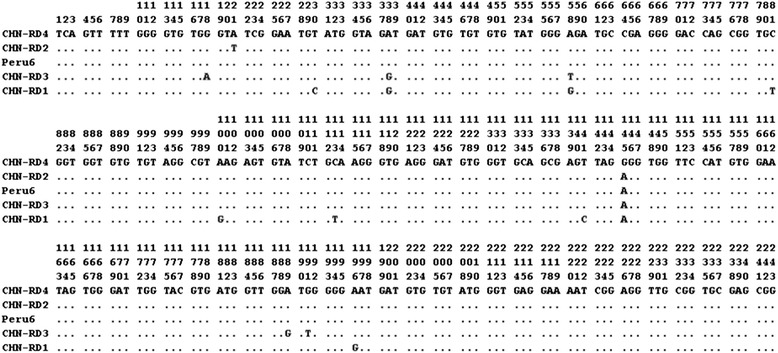
Sequence variation in the ITS region of the rRNA gene of *E. bieneusi* isolates. The ITS sequences of the known genotype Peru6 and four novel genotypes (CHN-RD1 to CHN-RD4) identified in this study were aligned with one another. Dots indicate the same base identity as the ITS gene sequence of genotype CHN-RD4

Five ITS genotypes of *E. bieneusi* were obtained in the three farms, including one known genotype Peru 6 (syn. PtEbI, PtEbVII) (*n* = 6) and four novel genotypes (GenBank: KR632538-KR632541) named as CHN-RD1 (*n* = 12) and CHN-RD2 to CHN-RD4 (one each) (Table 2). 13 nucleotide polymorphic sites were observed among them (Fig. 1). In fact, a high degree of genetic variability of deer-derived *E. bieneusi* isolates has been described in the ITS gene region of the rRNA gene [9, 11, 15]. To date, the number of genotypes of *E. bieneusi* in deer has increased to 29, including 19 genotypes (WL4, WL18, WL19, I, J, LW1 and DeerEb1-DeerEb13) in white-tail deer [11, 15], five genotypes (BEB6 and HLJD-I to HLJD-IV) in sika deer and one genotype (HLJD-V) in a red deer in China [9].

**Fig. 2:**
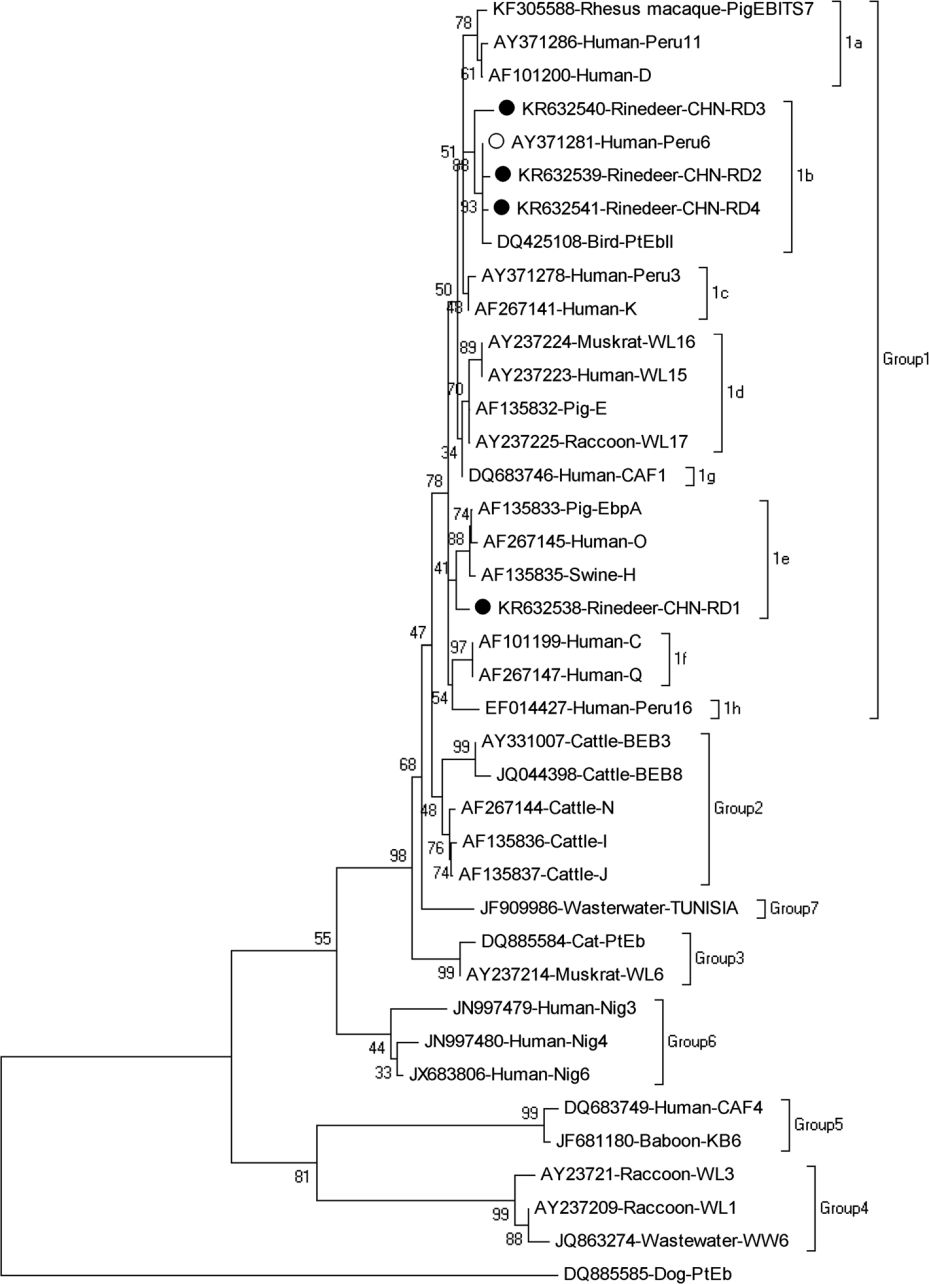
Phylogenetic relationship of *Enterocytozoon bieneusi* genotype groups. The relationship of *E. bieneusi* genotypes identified in the present study and other known genotypes deposited in GenBank was inferred by a neighbor-joining analysis of ITS sequences based on genetic distance by the Kimura two-parameter model. The numbers on the branches are percent bootstrapping values from 1000 replicates. Each sequence is identified by its accession number, host origin, and genotype designation. The group terminology for the clusters is based on the work of Karim et al. [25]. The solid and open circles indicate novel and known genotypes identified in this study, respectively

Genotype Peru 6 has been found in humans in Peru and Portugal [17–20], in mammals (cattle, sheep, goats, dogs) in USA, China and Portugal [16, 21, 22] and in birds (pigeons and a lovebird) in Portugal [23] as well as in wastewater in China [24]. The findings above suggest the reindeers infected with genotype Peru 6 may pose a threat to humans and other susceptible animal hosts. In phylogenetic analysis, all the novel genotypes belonged to group 1 previously described as a zoonotic group. Genotype CHN-RD1 was sub-clustered into 1e while genotypes CHN-RD2 to CHN-RD4 fell into 1b together with Peru 6 (Fig. 2), suggesting the potential for zoonotic transmission.

## Conclusion

This is the first report of *E. bieneusi* infection in reindeers, expanding the host range of *E. bieneusi.* The fact of genotype Peru 6 reported previously in humans and the finding of all the novel genotypes falling into zoonotic group 1 suggest the possibility of zoonotic transmission of *E. bieneusi* from reindeers to humans. However, due to the limited geographical distribution and the small population of reindeers as well as the fewer infected animals in China, public health significance of infected reindeers is relatively low. In spite of this, advice should be given to those people having close contact with reindeers.

## References

[1] Didie ES, Weiss LM (2006). Microsporidiosis: current status. Curr Opin Infect Dis.

[2] Matos O, Lobo ML, Xiao L (2012). Epidemiology of *Enterocytozoon bieneusi* infection in humans. J Parasitol Res.

[3] Thellier M, Breton J (2008). *Enterocytozoon bieneusi* in human and animals, focus on laboratory identification and molecular epidemiology. Parasite.

[4] Santin M, Fayer R (2011). Microsporidiosis: *Enterocytozoon bieneusi* in domesticated and wild animals. Res Vet Sci.

[5] Karim MR, Yu F, Li J, Li J, Zhang L, Wang R (2014). First molecular characterization of enteric protozoa and the human pathogenic microsporidian, *Enterocytozoon bieneusi*, in captive snakes in China. Parasitol Res.

[6] Santín M, Fayer R (2009). *Enterocytozoon bieneusi* genotype nomenclature based on the internal transcribed spacer sequence: a consensus. J Eukaryot Microbiol.

[7] Karim MR, Dong H, Yu F, Jian F, Zhang L, Wang R (2014). Genetic diversity in *Enterocytozoon bieneusi* from dogs and cats in China: host specificity and public health implications. J Clin Microbiol.

[8] Yang J, Song M, Wan Q, Li Y, Lu Y, Jiang Y (2014). *Enterocytozoon bieneusi* genotypes in children in Northeast China and assessment of the risk of zoonotic transmission. J Clin Microbiol.

[9] Zhao W, Zhang W, Wang R, Liu W, Liu A, Yang D (2014). *Enterocytozoon bieneusi* in sika deer (*Cervus nippon*) and red deer (*Cervus elaphus*): deer specificity and zoonotic potential of ITS genotypes. Parasitol Res.

[10] Henriques-Gil N, Haro M, Izquierdo F, Fenoy S, del Aguila C (2010). Phylogenetic approach to the variability of the microsporidian *Enterocytozoon bieneusi* and its implications for inter- and intrahost transmission. Appl Environ Microbiol.

[11] Guo Y, Alderisio KA, Yang W, Cama V, Feng Y, Xiao L (2014). Host specificity and source of *Enterocytozoon bieneusi* genotypes in a drinking source watershed. Appl Environ Microbiol.

[12] Sulaiman IM, Fayer R, Lal AA, Trout JM, Schaefer FW, Xiao L (2003). Molecular characterization of microsporidia indicates that wild mammals Harbor host-adapte*d Enterocytozoon* spp. as well as human-pathogenic *Enterocytozoon bieneusi*. Appl Environ Microbiol.

[13] Sak B, Petrzelkova KJ, Kvetonova D, Mynarova A, Shutt KA, Pomajbikova K (2013). Long-term monitoring of *Microsporidia*, *Cryptosporidium* and *Giardia* infections in western Lowland Gorillas (*Gorilla gorilla gorilla*) at different stages of habituation in Dzanga Sangha protected areas, Central African Republic. PLoS One.

[14] Němejc K, Sak B, Květoňová D, Hanzal V, Janiszewski P, Forejtek P (2014). Prevalence and diversity of *Encephalitozoon spp.* and *Enterocytozoon bieneusi* in wild boars (*Sus scrofa*) in Central Europe. Parasitol Res.

[15] Santin M, Fayer R (2015). *Enterocytozoon bieneusi*, *giardia*, and *Cryptosporidium* infecting white-tailed deer. J Eukaryot Microbiol.

[16] Buckholt MA, Lee JH, Tzipori S (2002). Prevalence of *Enterocytozoon bieneusi* in swine: an 18-month survey at a slaughterhouse in Massachusetts. Appl Environ Microbiol.

[17] Bern C, Kawai V, Vargas D, Rabke-Verani J, Williamson J, Chavez-Valdez R (2005). The epidemiology of intestinal microsporidiosis in patients with HIV/AIDS in Lima, Peru. J Infect Dis.

[18] Cama VA, Pearson J, Cabrera L, Pacheco L, Gilman R, Meyer S (2007). Transmission of *Enterocytozoon bieneusi* between a child and guinea pigs. J Clin Microbiol.

[19] Sulaiman IM, Bern C, Gilman R, Cama V, Kawai V, Vargas D (2003). A molecular biologic study of *Enterocytozoon bieneusi* in HIV-infected patients in Lima, Peru. J Eukaryot Microbiol.

[20] Lobo ML, Xiao L, Antunes F, Matos O (2012). Microsporidia as emerging pathogens and the implication for public health: a 10-year study on HIV-positive and -negative patients. Int J Parasitol.

[21] Santín M, Trout JM, Fayer R (2005). *Enterocytozoon bieneusi* genotypes in dairy cattle in the eastern United States. Parasitol Res.

[22] Lobo ML, Xiao L, Cama V, Stevens T, Antunes F, Matos O (2006). Genotypes of *Enterocytozoon bieneusi* in mammals in Portugal. J Eukaryot Microbiol.

[23] Lobo ML, Xiao L, Cama V, Magalhães N, Antunes F, Matos O (2006). Identification of potentially human-pathogenic *Enterocytozoon bieneusi* genotypes in various birds. Appl Environ Microbiol.

[24] Li N, Xiao L, Wang L, Zhao S, Zhao X, Duan L (2012). Molecular surveillance of *Cryptosporidium* spp., *Giardia duodenalis*, and *Enterocytozoon bieneusi* by genotyping and subtyping parasites in wastewater. PLoS Negl Trop Dis.

[25] Karim MR, Wang R, Dong H, Zhang L, Li J, Zhang S (2014). Genetic polymorphism and zoonotic potential of *Enterocytozoon bieneusi* from nonhuman primates in China. Appl Environ Microbiol.

